# The bryophyte community as bioindicator of heavy metals in a waterfall outflow

**DOI:** 10.1038/s41598-022-10980-9

**Published:** 2022-04-28

**Authors:** Narin Printarakul, Weeradej Meeinkuirt

**Affiliations:** 1 Department of Biology, Faculty of Science, Chiang Mai University, Chiang Mai, 50200 Thailand; 2 Research Center in Bioresources for Agriculture, Industry and Medicine, Chiang Mai University, Chiang Mai, 50200 Thailand; 3 Water and Soil Environmental Research Unit, Nakhonsawan Campus, Mahidol University, Nakhonsawan, 60130 Thailand

**Keywords:** Environmental sciences, Limnology

## Abstract

The species diversity and heavy metal accumulation in bryophytes were determined in Huay Pah Lahd stream in Doi Suthep-Pui National Park, Thailand. Eight bryophytes from two major taxonomic groupings (epilithic mosses and liverworts) were investigated. Of these, *Fissidens crispulus* var. *crispulus* was the most dominant taxon with an importance value (IV) of 28.98%, while *Ectropothecium zollingeri*, *Claopodium prionophyllum*, and *Hyophila involuta* were also dominant taxa with IV ≥ 10%. *Scopelophila cataractae*, a rare moss species with the lowest IV (0.91%) had the greatest capacity to accumulate metals in tissue, particularly Fe, Zn, Cd and Cu in protonemata (8026.7, 1187.2, 16.9 and 530.1 mg kg^−1^, respectively). The highest enrichment factors (EFs) of Zn, Cd and Cu (5.3, 2.4 and 0.9, respectively) were also found in *S. cataractae*, while the highest EF_Mn_ (1.1) was found in *H*. *involuta*. Enrichment factors of most heavy metals were < 5 from the study bryophytes, which suggests that natural processes were the key source of heavy metals. Dilution effects caused by increased water volume during the rainy season may be responsible for low pollutant loads and the maintenance of good water quality in this waterfall stream, which is favorable for biota and general environmental health.

## Introduction

Waterfalls may be caused by changes in sea-level, fault displacement, tectonic shifts, and other external factors. They occur at points in a rivers or streams where sudden steepening of a river channel, forces the water to flow vertically^1^. According to recent assessments, anthropogenic and natural activities are the principal sources of excessive heavy metal levels in waterfall streams. Increased heavy metal concentrations in waterfall streams have been related to chemical ingredients in cosmetic products (e.g., shampoos, powders, and toothpaste) carried into the forests by tourists^2^. However, output streams from waterfalls located primarily in highland national parks or other parcels of land designated as protected by governments are expected to have low heavy metal contamination. The physical condition of a waterfall habitat, with a high rate of flow and volume of water, tend to dilute heavy metal concentrations. Nonetheless rapid development in the tourism industry and various anthropogenic activities along waterfall streams have resulted in elevated concentrations of heavy metals in waterfall ecosystems in many locations in recent years^3^.

Limited investigations have focused on heavy metal contamination in waterfall streams due to their remote location from point sources of heavy metals. The monitoring of such pollutants in waterfall ecosystems, however, is necessary to ensure that they are safe zones and provide a haven for terrestrial and aquatic organisms^2^.

Bryophytes are significant species in aquatic and related terrestrial ecosystems, especially those where heavy metal concentrations have been elevated from anthropogenic sources. Heavy metals can be absorbed and accumulated by bryophytes through their surfaces from growth substrates such as soil and rock. Many bryophyte species have rhizoids, which are fine, short, filamentous root-like structures that can increase heavy metal cation exchange sites, thus making them suitable for monitoring deep soil pollution by heavy metals in aquatic habitats^4^. The aquatic moss *Leptodictyum riparium* (Hedw.) Warnt, for example, experiences high tolerance and accumulation of Cd. This taxon has been identified as an ecological bioindicator across a variety of pollution sources and environments^5^. This finding implies that the presence of certain bryophyte taxa may be used to monitor the ecological status of harsh environments.

In the present study, we explore species richness and diversity of bryophytes and the heavy metal accumulation capabilities of bryophytes occurring in the outflow from the Pah Lahd waterfall, Doi Suthep-Pui National Park, Thailand. Enrichment factors (EFs) were used to assess the contribution of the element in bryophyte biomass relative to anthropogenic sources.

## Results and discussion

### Physicochemical properties of waterfall stream

The environmental parameters of the Huay Pah Lahd waterfall stream are shown in Table 1. The water at the sampling site was shallow (0.4–1 m). Temperatures in water samples ranged from 20 to 33 °C, while water temperature along the bank of Pharadorn waterfall, located downstream of Romklao waterfall in Phu Hin Rong Kla National Park, was 25 °C^6^. While low water temperature and low light intensity are important for bryophyte development and influence primary productivity, nutrient enrichment in plant media (e.g., soil, sediment) is the most important factor for bryophyte growth^7^.

**Table 1:** Physicochemical properties of Pah Lahd stream.

Parameter	Unit	Value
Total hardness	mg L^−1^ CaCO^3^	31.7
Total solids	mg L^−1^	103
Fluoride (F^−^)	mg L^−1^	< 0.15
Ammonia–nitrogen (NH_3_^−^-N)	mg L^−1^	ND
Phosphate (PO_4_^3−^)	mg L^−1^	0.02
Chloride (Cl^−^)	mg L^−1^	6.2
Total nickel (Ni)	mg L^−1^	0.056
Nitrate-nitrogen (NO_3_^−^-N)	mg L^−1^	1.01
Total Kjeldahl nitrogen (TKN)	mg L^−1^	< 4.0
Total organic carbon (TOC)	mg L^−1^	ND
Biological oxygen demand (BOD)	mg L^−1^	< 1.0
Dissolved oxygen (DO)	mg L^−1^	5.03
Temperature	°C	25.3
pH		6.75
Depth	m	0.4–1

The quantities of nitrate-nitrogen (NO_3_-N), total Kjeldahl nitrogen (TKN) and phosphate (PO_4_^3−^) in the waterfall stream were 1.01, < 4.0, and 0.02 mg L^−1^, respectively, while ammonia–nitrogen (NH_3_-N) was undetectable. Excessive concentrations of phosphorus and nitrogen compounds, which are limiting factors and essential nutrients for aquatic life, can cause eutrophication. However, dilution effects during the rainy season in lotic ecosystems are a key factor in causing low nutrient concentrations in water bodies^8^. Rainfall strongly affected the physicochemical properties of the water because flow rate, depth, and water level, and nutrient run-off (and/or pollutant run-off) changed during the sampling period in the May to October Southwest Monsoon rainy season. During rainy periods in 2020, total rainfall and mean temperature in Chiang Mai Province were approximately 3475 mm and 28.8 °C, respectively^9^. While most heavy metal concentrations (Cu, Cd, Zn, Fe, Cr, Pb and Mn) in water samples were relatively low to undetectable, Ni was detected at 0.056 mg L^−1^. Furthermore, F^−^ and Cl^−^ contents were < 0.15 and 6.2 mg L^−1^, respectively, which are considered low according to permissible limits set by the World Health Organization (WHO) at 0.6–1.5 mg L^−1^ and 250 mg L^−1^, respectively^10^. Total organic carbon (TOC) content of the water sample was below the detection limit (< 0.05 mg L^−1^), which is due to the low level of hydrocarbon contaminants in the water sources^11^.

Total solids content is a direct measurement of the total mass of organic and inorganic particles suspended in water, as well as total dissolved ions^12^. A high total solids content in waters is the most likely cause of increased total hardness. Other possible sources of increased hard water content include Ca, Mg and heavy metals widely distributed in rocks and sediments^13^. The water in Pah Lahd stream was naturally soft (total hardness 31.7 mg L^−1^) with very low total solids and minerals. Soft water is defined as a water sample having a low amount of calcium carbonate^14^.

The pH value of the studied stream was near neutral (≈ 6.8). Slightly acidic water (~ pH 5–6) occurs naturally, enabling some heavy metals that are adsorbed to mineral surfaces to be dissolved in aquatic ecosystems, as well as enhancing mineral dissolution in sediment^15^. A pH range of 6.5 to 9.0 provide acceptable protection for survival of freshwater biota, implying that there is no detriment to the ecosystem or biota in the current study^16^. The DO level of the study site (5.03 mg L^−1^) was nearly equivalent to the DO water quality standard in a Thai waterfall stream (6 mg L^−1^)^17^; nevertheless, a reduction in DO levels might be linked to human activities, i.e., the presence of relatively large numbers of tourists and improper waste disposal^17^. High flow velocity and turbulence of a waterfall increases DO content^18^. Biological oxygen demand (BOD) is another important indicator of water quality, as it measures the quantity of oxygen required for microbial respiration and biological degradation of organic matter in water. Reduced BOD levels imply that the quantity of organic substances is promoting the growth of microbial populations, thus enhancing the available DO content for aquatic life^19^. The water sample in the current study had a low BOD level (< 1 mg L^−1^), indicating that the waterfall ecosystem had good water quality.

### Bryophyte taxa in the study site

A total of eight bryophytes were collected from different microhabitats and life modes. These constituted two major taxonomic groupings (1) mosses: epilithic or rupicolous mosses (three acrocarpous mosses: *Hyophila involuta* (Hook.) A. Jaeger (Pottiaceae), *Scopelophila cataractae* (Mitt.) Broth. (Pottiaceae) and *Bryum* sp. (Bryaceae); three aquatic mosses (one acrocarpous moss, *Fissidens crispulus* Brid. var. *crispulus* (Fissidentaceae) and two pleurocarpous mosses: *Claopodium prionophyllum* (Müll. Hal.) Broth. (Leskeaceae), and *Ectropothecium zollingeri* (Müll. Hal.) A. Jaeger (Hypnaceae); and (2) liverworts: one thalloid liverwort, *Marchantia emarginata* Reinw. Blume & Nees var. *emarginata* (Marchantiaceae); and one leafy liverwort: *Porella acutifolia* (Lehm. & Lindenb.) Trevis. var. *birmanica* S. Hatt. (Porellaceae).

**Figure 1:**
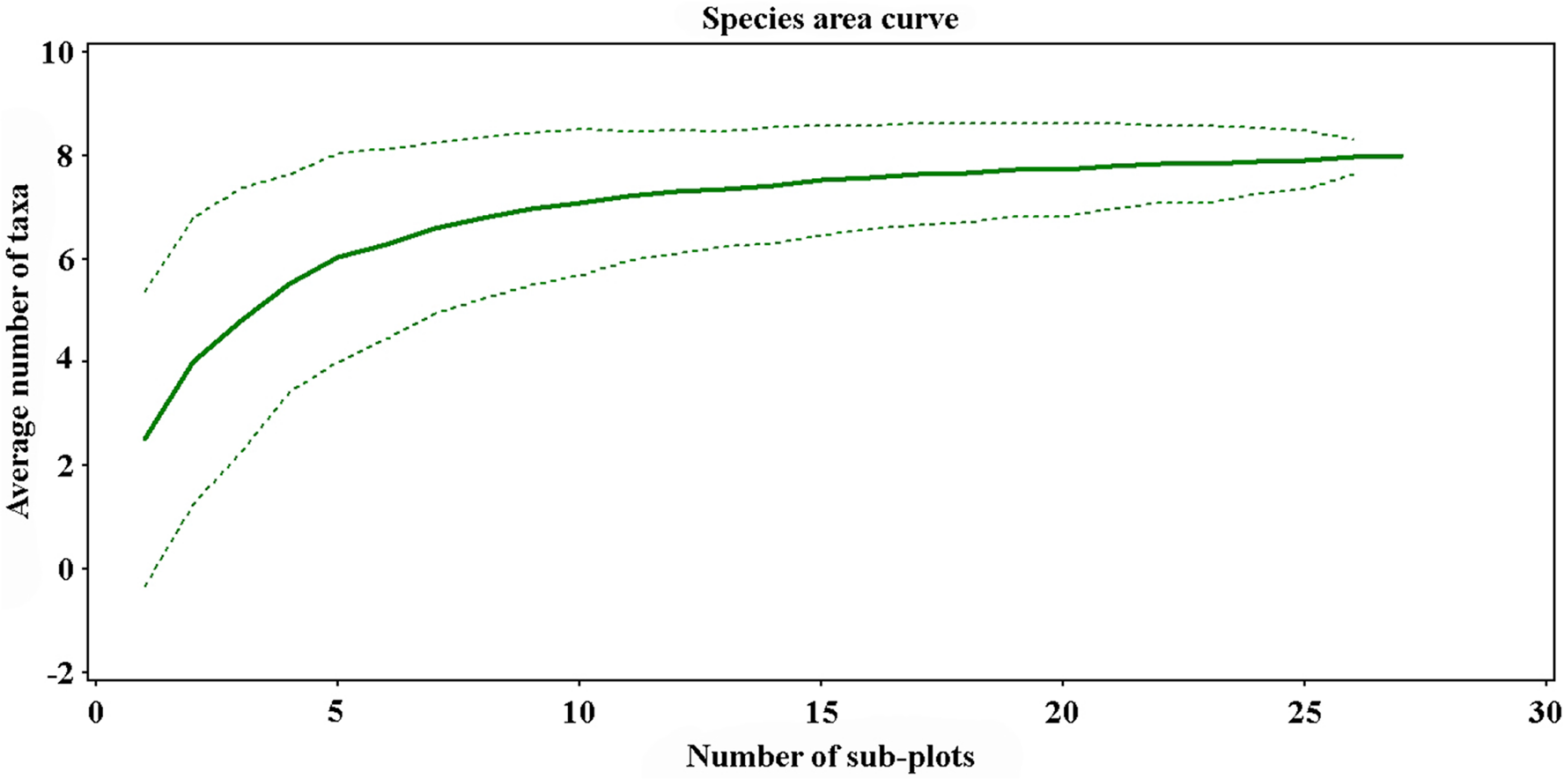
Species area curve showing relationship in numbers of subplot and numbers of species of bryophytes in Huay Pah Lahd stream, Doi Suthep-Pui national park.

**Figure 2:**
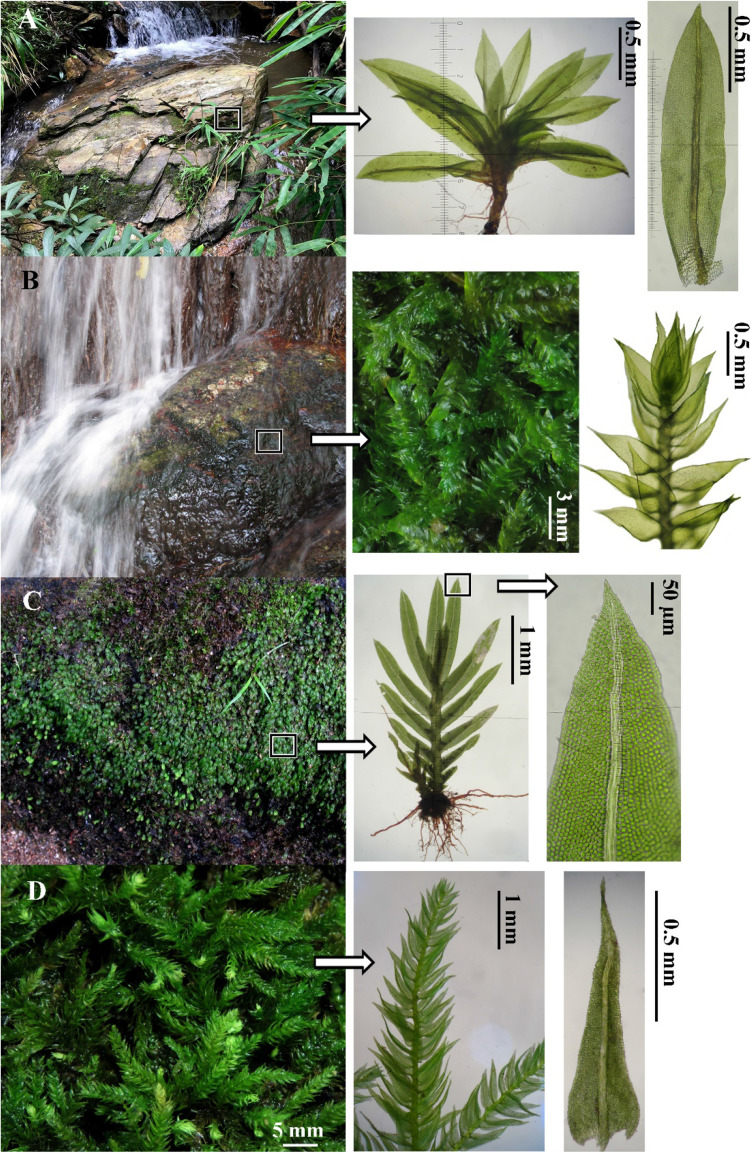
Rare species (**A**) and dominant taxa (**B-D**) of mosses grown on wet rocks in Huay Pah Lahd stream outflow, Doi Suthep-Pui national park: (**A**) *S. cataractae*; (**B**) *E. zolligeri*; (**C**) *F. crispulus* var. *crispulus*; and (**D**) *C. prionophyllum*.

The species area curves showed a smooth curvature, but did not reach an asymptote (Fig. 1). The sampling completeness (approximately 80.8%) also indicated that 27 subplots from the three 10-m plots were adequate for estimating the bryophyte diversity in the Huay Pah Lahd stream (Table 2 and Fig. 2). According to the Jackknife estimate of the total number of taxa, the number of expected species for the community was between 7 and 10. Furthermore, bryophyte diversity and coverage in the three study plots showed similar patterns (Table 2). Plot 3 had the lowest species richness (5 taxa) and Shannon–Wiener diversity index (1.31), whereas Plot 1 had the highest species richness (8 taxa) and Shannon–Wiener diversity index (1.67). The Pielou’s evenness index was relatively similar among the plots, ranging from 0.79 to 0.81 (total = 0.54), whereas the coverage of bryophytes in the three study plots ranged from 48.54 to 53.85%. Despite being sampled in the same season (wet), habitat heterogeneity and differing environmental conditions among the three plots cause differences in the composition and structure of bryophytes.

**Table 2:** Bryophyte diversity of three sampling plots (10-m^2^ per plot) along Huay Pah Lahd stream, Doi Suthep-Pui national park.

Plots	Numbers of subplot use	Numbers of observed taxa (species richness, *s*)	Jackknife’s estimation total numbers of taxa	Shannon-Weiner index (*H*′)	Pielou’s evenness index *(J*′*)*	Total coverage of bryophytes (%)	Sampling completeness (%)
Plot 1	9	8	8.9	1.67	0.81	53.67	89.88
Plot 2	11	6	7.0	1.43	0.79	48.54	85.71
Plot 3	7	5	7.6	1.31	0.81	53.85	65.78
Total	27	8	9.9	1.13	0.54	51.63	80.80

A previous study in forest (Kanghe Provincial Nature Reserve, Guangdong Province, China) revealed a significantly higher number of ground bryophytes (37 taxa) and a Shannon-Weiner index value of up to 2.9, whereas the present study in the waterfall stream found a significantly lower number of bryophytes (4.6 times) and Shannon-Weiner index value (2.6 times), respectively^20^. To some extent, the specialized habitat of bryophytes, such as cave, has been discovered, with a similar number of species and diversity index to this study; however, it had lesser bryophyte coverage because light intensity is a limiting factor in caves, which may affect plant growth and development^21^.

There were three dominant lithophytic mosses, which are aquatic, semi-aquatic, or found on soil and rock near seasonally dried streams of the Huay Pah Lahd waterfalls. These bryophytes can survive on muddy, debris-covered rocks, which are flooded during the rainy season and which dry up during the hot-dry season (February to April), viz. *E. zollingeri*, *F. crispulus* var. *crispulus*, and *C. prionophyllum* (Fig. 2B-D). Of the eight observed bryophyte taxa (Table 3), *Fissidens crispulus* var. *crispulus* was the most dominant taxon in the waterfall stream, with an importance value (IV) of 28.98%, relative frequency (RF) of 20.59%, and relative cover (RC) of 37.37% (Fig. 2C). This moss taxon is a rheophyte that can be found in wet rocks of waterfall streams throughout Indochina during the rainy season^22^. Other dominant bryophyte taxa with IV ≥ 10% include *E. zollingeri*, *C. prionophyllum*, and *H. involuta* (Table 3 and Fig. 2B, D). These moss taxa can be found in various moist or semi-wet locations, including waterfall ecosystems^23,24^. Furthermore, *S. cataractae* can be regarded a rare moss species because it had the lowest IV (0.91%), and was found in only one subplot with an extremely low value of relative cover (RC = 0.36%) (Table 3 and Fig. 2A). *Scopelophila cataractae* is a rare species found in Thailand that is listed as endangered species in the IUCN Red List’s threatened category^25,26^. *Scopelophila cataractae* is found in various parts of the world including China, Korea, Japan, Papua New Guinea, and North and South America^27,28^. A protonemal colony of *S. cataractae* was observed at the same time (June, during the rainy season) and same study site as in the previous study^26^. Narrow, light-green patches of the protonemata occur along the wet rocks (*c.* 50 cm height) in streams, together with colonies of other bryophytes such as *H. involuta*. The colony consisted of numerous filamentous protonema which produced shoots of *S. cataractae* with multicellular gemmae in the axils of young leaves.

**Table 3:** Bryophyte community composition and structure of Huay Pah Lahd stream, Doi Suthep-Pui national park.

Species	Family	F	RF (%)	AC	RC (%)	IV	IV (%)
1. *Bryum* sp.	Bryaceae	5	7.35	0.74	1.43	8.79	4.39
2. *C. prionophyllum*	Leskeaceae	10	14.71	8.96	17.36	32.07	16.03
3. *E. zollingeri*	Hypnaceae	16	23.53	14.89	28.84	52.37	26.18
4. *F. crispulus* var. *crispulus*	Fissidentaceae	14	20.59	19.30	37.37	57.96	28.98
5. *H. involuta*	Pottiaceae	13	19.12	4.22	8.18	27.30	13.65
6. *M. emarginata* var. *emarginata*	Marchantiaceae	5	7.35	1.19	2.30	9.65	4.82
7. *P. acutifolia* var. *birmanica*	Porellaceae	4	5.88	2.15	4.16	10.04	5.02
8. *S. cataractae*	Pottiaceae	1	1.47	0.19	0.36	1.83	0.91

F = frequency; RF = relative frequency; AC = average percent cover; RC = relative cover; IV = importance value.

*Porella acutifolia* var. *birmanica* was first discovered in Burma and is widespread in Burma, Vietnam, Laos, and Thailand^29,30^. This taxon has been reported from Doi Suthep and Ru See (Hermit) cave in Doi Suthep-Pui National Park, at elevations of about 1100–1200 m^29^. *Hyophila involuta*, *Bryum* sp., and *M. emarginata* may be termed pioneer taxa in open and urban environments. *Hyophila involuta* occurs in a very wide variety of habitats including deserts, humid region soil, wet rocks, and waterfall stream banks, an even as on concrete structures in urban settings^24,31,32^. Unfortunately, the *Bryum* sp. specimen lacked sporophyte materials; thus, it was not possible to identify it to the species level. Members of the Bryaceae family, on the other hand, are abundant in urban and disturbed regions across the world, and can be seen growing with potted plants^33^. *Marchantia emarginata* var. *emarginata*, a cosmopolitan taxon of thalloid liverwort^34^, is abundant in soils and on rocks near stream banks and other locations in Chiang Mai Province.

### Heavy metal concentrations in bryophyte tissues and substrates

Bryophytes, which lack true roots and have a thick cuticle, take advantage of a high surface/volume ratio and a high cation-exchange capacity, allowing capillary action to transport available minerals and water to the entire surface^35^. Furthermore, phyllids (leaf-like structures) and thalli of bryophytes have highly absorbent surfaces and an absence of waxy cuticle over the laminal surfaces. As a consequence, cell walls readily absorb moisture and a wide range of minerals and metal ions from water that flows over the plant^36^. Among our specimens (Table 4), Cu levels in tissues ranged from 8.5 mg kg^−1^ (*P*. *acutifolia* var. *birmanica* gametophyte) to 530.1 mg kg^−1^ (*S*. *cataractae* protonema); Cd from 4.8 mg kg^−1^ (*E*. *zollingeri* gametophyte) to 16.9 mg kg^−1^ (*S*. *cataractae* protonema); Zn from 129.4 mg kg^−1^ (*H*. *involuta* gametophyte) to 1187.2 mg kg^−1^ (*S*. *cataractae* protonema); Fe from 3962.5 mg kg^−1^ (*H*. *involuta* gametophyte) to 8026.7 mg kg^−1^ (*S*. *cataractae* protonema); and Mn from 143.3 mg kg^−1^ (*S*. *cataractae* gametophyte) to 504.6 mg kg^−1^ (*C*. *prionophyllum* gametophyte).

**Table 4:** Heavy metal accumulation in bryophyte tissues (*n* = 3).

Families	Botanical names	Plant part	Heavy metal accumulation (mg kg^−1^)				
			Cu	Cd	Zn	Fe	Mn
Pottiaceae	*S. cataractae*	Gametophyte (without protonema)	506.0 ± 0.6b	9.2 ± 0.1c	846.1 ± 48.0b	5434.3 ± 42.6de	144.3 ± 3.5d
Pottiaceae	*S. cataractae*	Protonema	530.1 ± 25.8a	16.9 ± 0.5a	1187.2 ± 393.6a	8026.7 ± 164.0a	144.5 ± 0.0d
Porellaceae	*P. acutifolia* var. *birmanica*	Gametophyte	8.5 ± 2.3d	12.0 ± 1.8b	161.3 ± 2.8c	6877.6 ± 479.5b	383.3 ± 36.9c
Pottiaceae	*H. involuta*	Gametophyte	9.6 ± 1.9d	5.2 ± 0.6d	129.4 ± 4.6c	3962.5 ± 146.5f.	467.2 ± 12.0ab
Marchantiaceae	*M. emarginata* var. *emarginata*	Thallus	23.7 ± 1.4d	8.7 ± 0.3c	203.2 ± 17.4c	4264.7 ± 111.9f.	341.9 ± 23.6c
Fissidentaceae	*F. crispulus* var. *crispulus*	Gametophyte	24.2 ± 4.0d	8.2 ± 0.8c	197.6 ± 0.2c	5386.0 ± 171.3e	448.6 ± 25.2b
Leskeaceae	*C. prionophyllum*	Gametophyte	10.3 ± 3.2d	8.2 ± 2.0c	226.9 ± 26.6c	6370.0 ± 371.6c	504.6 ± 22.9a
Hypnaceae	*E. zollingeri*	Gametophyte	18.2 ± 3.0d	4.8 ± 1.0d	140.9 ± 8.3c	4391.3 ± 87.5f.	354.2 ± 18.4c
Bryaceae	*Bryum* sp.	Gametophyte	135.3 ± 21.2c	6.2 ± 1.3d	225.5 ± 16.7c	5869.9 ± 273.6d	482.6 ± 66.1ab

For each parameter, values followed by different letters indicate significant difference at 5% probability level.

*Cu* copper, *Cd* cadmium, *Zn* zinc, *Fe* iron, *Mn* manganese.

The gametophytes of *S*. *cataractae* had considerably greater Cu accumulation (*p* < 0.05) or approximately 3.7–59 times greater than among other bryophytes, although protonema of *S*. *cataractae* had an even higher Cu concentration (*p* > 0.05). Because it accumulated Cu primarily in gametophyte tissue, *S*. *cataractae* is categorized as a hyperaccumulator of Cu^37^. Copper is an essential nutrient that is required for plant development and growth. It plays a significant role in regulating physiological functions such as the photosynthetic and respiratory electron transport chains, nitrogen fixation, protein metabolism, antioxidant production, the ROS defense system, cell wall metabolism, and hormone perception, and acts as an essential cofactor for numerous metalloproteins^38^. At the cellular level, however, excessive Cu concentrations are harmful to plants due to inactivation and disruption of enzyme activity or protein functions^38^. Gametophytes of *S*. *cataractae* accumulated substantial quantities of Cd, Zn and Fe, with concentrations of 9.2 mg kg^−1^, 846.1 mg kg^−1^ and 5434.3 mg kg^−1^, respectively. *Scopelophila cataractae* has been shown to accumulate substantial amounts of heavy metals such as Cd, Cu and Zn in contaminated soil (e.g., Cu tailings)^39^. Remarkably low Cu concentrations were detected in *C. prionophyllum* and *H*. *involuta* (10.3 kg^−1^ and 9.6 mg kg^−1^, respectively).

Highest Cu concentrations were measured in sediment substrate of shoot colonies and protonemal colonies of *S. cataractae* (251.6 mg kg^−1^ and 239.4 mg kg^−1^, respectively) (*p* < 0.05), whereas substantial Fe concentrations were found in sediment substrate of gametophyte colonies of *H. involuta* (3127.1 mg kg^−1^) (*p* < 0.05) and sediment substrate of protonemal colonies, shoot colonies and decayed moss of *S. cataractae* (2345.3, 2289.4 and 1963.7 mg kg^−1^, respectively) (Table 5)*.* Copper concentrations in substrates of *S*. *cataractae* and water were generally in the following order: sediment substrate > rock > water. According to recent research, growth substrate is a key source of heavy metals in stream environments; this may have led to increased absorption and accumulation of Al, Cu and Zn in leaf surfaces and protonemata of *S*. *cataractae* gametophytes^26^. Cadmium and Zn concentrations in rock substrates of *S. cataractae* and *P. acutifolia* var. *birmanica* were low (0.5 and 0.3 mg kg^−1^ for Cd, and 34.9 and 31.2 mg kg^−1^ for Zn, respectively). Heavy metal (Cu, Fe and Mn) concentrations in rock substrates of *P. acutifolia* var. *birmanica* were similar to those of rock substrates of *S. cataractae*. Both were located in similar environments and so received heavy metals from similar sources and mechanisms.

**Table 5:** Heavy metal accumulation in bryophyte substrates (*n* = 3).

Material	Heavy metal accumulation (mg kg^−1^)				
	Cu	Cd	Zn	Fe	Mn
Decayed Cu moss	188.4 ± 8.4b	1.9 ± 0.3a	80.5 ± 5.7b	1963.7 ± 99.7bc	246.9 ± 2.3ab
Sediment substrate of shoot colony of *S. cataractae*	251.6 ± 35.2a	1.5 ± 0.1bcd	59.3 ± 4.3c	2289.4 ± 363.3b	143.9 ± 28.9cd
Sediment substrate of protonemal colony of *S. cataractae*	239.4 ± 1.4a	1.7 ± 0.3ab	65.9 ± 6.3c	2345.3 ± 298.4b	126.4 ± 3.0d
Rock substrate of *S. cataractae*	56.3 ± 4.3c	0.5 ± 0.2f	34.9 ± 1.3d	1259.9 ± 16.9d	243.4 ± 38.6bc
Rock substrate of *P. acutifolia* var. *birmanica*	51.2 ± 15.6c	0.3 ± 0.2f	31.2 ± 4.7d	1234.9 ± 40.1d	202.6 ± 76.6bc
Sediment substrate of shoot colony of *H. involuta*	11.2 ± 0.9d	1.7 ± 0.2abc	122.6 ± 0.7a	3127.1 ± 312.0a	314.5 ± 8.5a
Sediment substrate of shoot colony of *M. emarginata* var. *emarginata*	55.6 ± 1.7c	1.2 ± 0.0de	66.2 ± 1.2c	1174.3 ± 2.1d	297.7 ± 12.9a
Sediment substrate of shoot colony of *F. crispulus* var. *crispulus*	57.5 ± 4.2c	1.2 ± 0.1de	66.8 ± 3.1c	1172.8 ± 8.1d	312.0 ± 14.3a
Sediment substrate of shoot colony of *C. prionophyllum*	54.5 ± 4.1c	1.2 ± 0.1de	64.9 ± 3.3c	1164.5 ± 16.3d	301.4 ± 18.4a
Sediment substrate of shoot colony of *E. zollingeri*	60.8 ± 0.4c	1.3 ± 0.0cde	76.3 ± 5.8b	1663.7 ± 99.8c	312.0 ± 23.5a
Sediment substrate of shoot colony of *Bryum* sp.	56.5 ± 6.3c	1.0 ± 0.1e	62.7 ± 0.8c	1156.6 ± 12.3d	273.3 ± 29.3ab

For each parameter, values followed by different letters indicate significant difference at 5% probability level.

*Cu* copper, *Cd* cadmium, *Zn* zinc, *Fe* iron, *Mn* manganese.

Heavy metal localization in bryophyte tissues implies the suitability of these organisms for biomonitoring freshwater pollution^40,41^. The accumulation potential of heavy metals in cell walls of bryophytes can be linked to degree of tolerance and resistance to heavy metals. The substantial heavy metal concentrations that bind to pectin is considered to be an effective defensive mechanism against divalent metal cations such as Cu^2^^+^ and Pb^2^^+^, allowing for successful adaptation to heavy metal-enriched substrates^41^.

Many bryophyte taxa have been tested for their tolerance and accumulation capabilities at both laboratory and field scales. For example, *Bryum radiculosum* Brid. (Bryaceae) grown in industrial areas of Portoscuso (Sardinia, Italy) has been used as bioindicator for trace elements such as Pb, Cd and Zn, with accumulation rates at 61–2141 mg kg^−1^, 3–40.6 mg kg^−1^ and 32–2360 mg kg^−1^, respectively^35^. *Bryum radiculosum* from a previous study that grew in locations exposed to heavy metals from Cu-containing pesticides in vineyards. This moss taxon accumulated considerably lower amounts of Cu than *Bryum* sp. in the present study (135.3 mg kg^−1^), or less than 1.4–13.5-fold^42^. Furthermore, *P. acutifolia* var. *birmanica*, *C. prionophyllum* and *Bryum* sp. accumulated substantial Fe (6877.6 mg kg^−1^, 6370 mg kg^−1^ and 5869.9 mg kg^−1^, respectively), and Cd (12 mg kg^−1^, 8.2 mg kg^−1^ and 6.2 mg kg^−1^, respectively), and modest amounts of Zn (161.3 mg kg^−1^, 226.9 mg kg^−1^ and 225.5 mg kg^−1^, respectively). Cadmium is a hazardous metal; Cd exposure in moss media at 10 μM inhibited photosynthesis and caused nutrient deficiencies, leading to chlorosis in gametophyte tissues of *Physcomitrium patens* (Hedw.) Mitt. (Funariaceae) and aquatic moss, *Fontinalis antipyretica* Hedw. (Fontinalaceae)^43^. Zinc and Fe are major components of numerous enzymes and proteins in plants and are thus essential micronutrients for biota. High concentrations of Zn and Fe, on the other hand, can be toxic to moss cells, affecting the entire plant by decreasing moss growth and development^44^.

In the present study, substantial Mn concentrations were detected in gametophytic tissues of *C. prionophyllum*, *Bryum* sp., *H*. *involuta* and *F. crispulus* var. *crispulus* (504.6 mg kg^−1^, 482.6 mg kg^−1^, 467.2 mg kg^−1^ and 448.6 mg kg^−1^, respectively). Manganese accumulation in the study bryophytes were much higher (144.3–504.6 mg kg^−1^) compared to those in four moss taxa *Bryum argenteum* Hedw. (Bryaceae), *Bryum capillare* Hedw. (Bryaceae), *Brachythecium* sp. (Brachytheciaceae), and *Hypnum cupressiforme* Hedw. (Hypnaceae) grown in various locations (roadside, populated areas, forests, croplands); Mn accumulation (0.1–8.6 mg kg^−1^)^45^. Excessive Mn concentration in plant tissues can induce oxidative stress, alter enzymatic activity, absorption and accumulation of nutrients, and translocation of certain elements including calcium (Ca), magnesium (Mg), Fe and phosphorus (P)^46^.

The rediscovery of *P. acutifolia* var. *birmanica* in Huay Pah Lahd stream after a half-century^29^ may suggest that the Doi Suthep-Pui National Park still serves as a haven for sensitive bryophytes, and is minimally affected by anthropogenic activities and thus can support a suitable habitat for bryophytes. Nonetheless, anthropogenic activities around the sampling location may have introduced heavy metals into sediment and water, increasing heavy metal absorption and accumulation in bryophyte tissue^26^. Because *P. acutifolia* var. *birmanica* also accumulated substantial heavy metals, particularly Cd, the presence of this leafy liverwort may be used as bioindicator in future research for monitoring changes in environmental patterns of stream ecosystems.

### Enrichment factors of heavy metals

**Figure 3:**
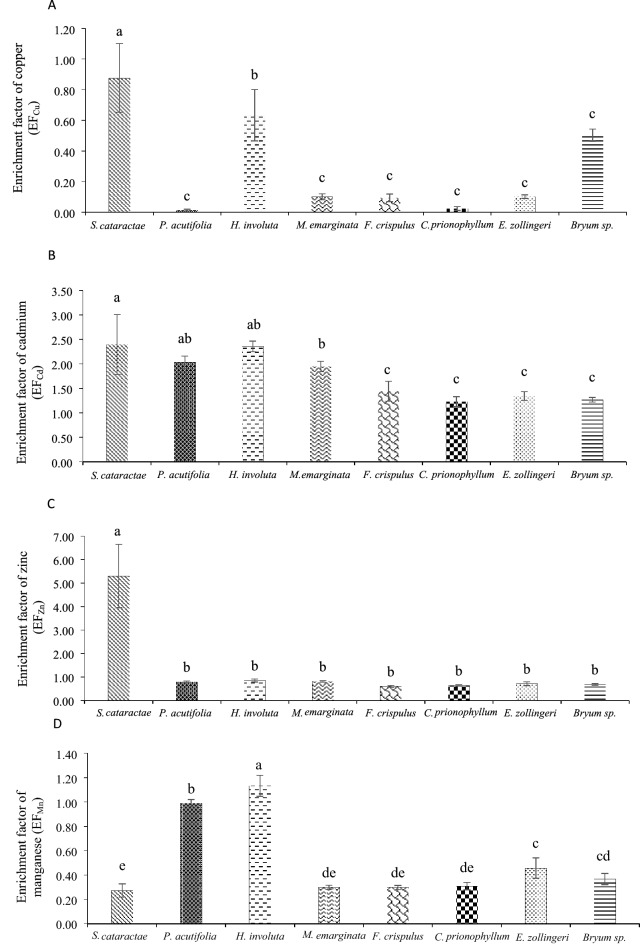
Means for the enrichment factor (EF) of Cu, Cd, Zn and Mn (**A-D**) with corresponding standard deviations (SD) for four elements in bryophytes relative to the sediment substrates.

The EFs of heavy metals were generally in the following order: Cd > Zn > Mn > Cu (Fig. 3). Significant EFs of all the studied heavy metals (5.3, 2.4 and 0.9 for EF_Zn_, EF_Cd_ and EF_Cu_, respectively) were found in *S. cataractae* (*p* < 0.05), except for EF_Mn_ (0.3). A previous study found that EFs of Cu, Cd, and Zn increased as the quantities of heavy metals in moss tissue increased^47^. This was not the case in the current study, however. This finding could be due to differences in moss tolerance and adaption^35^. The fact that EFs < 5 for study plants indicates that the sources of these metals are lithologic, i.e., sediment, water, and rock^40^. Furthermore, the EF_Zn_ > 5 for *S. cataractae* indicated that Zn contamination at the sampling site ranged between low and moderate. Zinc is readily absorbed by plants in high quantities due to its abundance in the lithosphere as well as its moderate solutability, but it is rarely toxic to plants^48^. The EF of Mn for *H. involuta* was low, but nevertheless had the highest value (1.1; *p* < 0.05), followed by *P. acutifolia* (1.0). According to Ali Hussen et al.^3^, tourist activities are now regarded as key sources of heavy metals in waterfall streams, the resultant water contamination often exceeding the standard for drinking water. Because the study site was close to hill-tribe villages and tourist sites, pollutant contamination in waterfall steam from anthropogenic activities was likewise expected.

Each bryophyte taxon has specific habitat and environmental preferences and a unique ecological niche. These factors, in combination with their sensitivity to environmental change, and propensity to take up and accumulate pollutants from soil and water, and accumulate trace metals (Cd, Pb, Ni and Cr) from the atmosphere, soil and water in contaminated areas worldwide make bryophyte taxa useful indicators of vegetation alteration and climate change and the overall health status of habitats^49,50^. To date, few studies exist which investigate bryophytes as bioindicators of heavy metals in both terrestrial and aquatic environments in Thailand.

## Conclusions

The species diversity and heavy metal accumulation potential of bryophytes present in a waterfall stream at Doi Suthep-Pui National Park, northern Thailand, were investigated for the first time. A total of 8 bryophyte taxa was identified. Of these, the most dominant taxon was *F. crispulus* var. *crispulus*, an aquatic moss, while *S. cataractae*, an epilithic moss, was rare. *Scopelophila cataractae* was the best bioindicator in the waterfall stream because it had the highest accumulation potential of heavy metals such as Cu, Cd, Zn and Fe concentrations, and the highest EFs of Cu, Cd and Zn. Bryophytes from the study site were enriched with low concentrations of heavy metals, as demonstrated by the EFs of heavy metals (< 5). Furthermore, the presence of substantial EF_Zn_ in the dominant moss, *H. involuta*, indicates that heavy metals were obtained in part from anthropogenic activities, e.g., nearby tourist and community use. While the environmental variable values in the waterfall stream were presently within acceptable concentrations for biological function maintenance in this healthy natural park ecosystem buffer environment, they may be destined to rise with increased human use.

## Methods

### Study site

Doi (Mountain) Suthep*,* Doi Suthep-Pui National Park, Thailand, is the source of several waterfalls. Tourist activities have grown recently in the area of the Huay Pah Lahd waterfall. On the eastern slope of Doi Suthep at *c.* 580–660 m elevation (18° 47′ 56.0′′ N, 98° 55′ 52.0′′ E), the waterfall is surrounded by mixed evergreen/deciduous forest in small villages that receive tourists. Stream depth ranges from 0.2 to 0.6 m. In 2020 total annual rainfall, mean relative humidity and mean temperature in Chiang Mai Province were approximately 1085.1 mm, 63.4% and 27.6 °C, respectively. Anthropogenic activity was suspected to be a major cause of water pollution in the waterfall streams.

### Collection of plant, sediment and water samples

During a field excursion on June 15, 2020, conducted with authorization from the Department of National Parks, Wildlife, and Plant Conservation of Thailand. Bryophyte specimens were collected in the Huay Pah Lahd stream with a plastic spatula on wet rocks and the thin soil layer upon the rock, which serve as substrates for all bryophyte taxa. Individual plant samples were stored in clean plastic bags, labeled, and transported promptly to the laboratory in an ice-filled box. Plant tissue was rinsed with deionized (DI) water for 30 s to remove excess soil, visible debris, fine stones and pebbles, loosely attached mineral particles, and tiny organic materials, then air-dried at room temperature and stored at 4 °C until required^51^. The plant material used in this study was formally identified by Narin Printarakul, Department of Biology, Faculty of Science, Chiang Mai University. Dried plant specimens i.e., *H. involuta* (Printanakul N. 15062020_1); *S. cataractae*; (Printanakul N. 15062020_2); *Bryum* sp. (Printanakul N. 15062020_3); *F. crispulus* var. *crispulus* (Printanakul N. 15062020_4); *C. prionophyllum* (Printanakul N. 15062020_5); *E. zollingeri* (Printanakul N. 15062020_6); *M. emarginata* var. *emarginata* (Printanakul N. 15062020_7); *P. acutifolia* var. *birmanica* (Printanakul N. 15062020_8), were preserved and deposited in the Chiang Mai University (CMUB) Herbarium, which is a publicly accessible facility at Chiang Mai University.

Bryophyte taxa identification and morphological characteristics were investigated using Olympus stereo (SZ-30) and compound (Eclipse E-200) microscopes with magnifications of 40 × and 40 × to 400x, respectively. The distinctive characteristics of bryophyte taxa were illustrated with light microscope (LM) photographs by using a Nikon (D7000) camera. Taxonomic identification of bryophyte taxa followed Eddy^31,52^, Gradstein^53^, Hattori^30^, Li et al.^27^, Wu et al.^54^ and Zhang and He^55^. Under a compound microscope, moss protonemata was harvested with forceps.

Soil samples of 0.5 m thickness were collected under bryophyte patches with a plastic spatula. Water samples were collected from the stream near the collection site in a 1 L polyethylene acid-washed bottle and stored in an ice-filled container (4 °C). In the field, pieces of rocks under bryophyte patches (only *S*. *cataractae* and *P. acutifolia* var. *birmanica*) were crushed using a hammer and stored in self-locking polyethyene bags and sealed in double bags before being transported to the laboratory. Collection of plant specimens was carried out in accordance with relevant institutional, national, and international guidelines and legislation.

### Physicochemical properties of water sample

Selected environmental parameters in the water sample were analyzed using the methodologies provided in APHA, AWWA, and WEF^56^, i.e., total solids, total hardness, NO_3_- N, NH_3_- N, TKN, PO_4_^3−^^3^, and BOD_5_. Total organic carbon (TOC) was determined using a TOC analyzer (multi N/C 2100/2100s, Analytik Jena, Germany), fluoride (F^−^) concentration with an ion selective electrode (Thermo Scientific, ORION STAR A324), and chloride (Cl^−^) with a Dionex ICS-900 ion chromatograph (Thermo Fisher Inc., Japan). Each water sample (50 mL) was passed through a cellulose membrane filter, 0.45 μm pore size, and then acidified with 0.05 mL double-distilled hydrochloric acid (HCl, Merck) to pH < 2. Heavy metals (i.e., Cd, Cu, Ni, Cr, Zn, Pb, Fe, and Mn) were determined by flame atomic absorption spectrophotometry (FAAS; AAnalyst200, PerkinElmer).

Water temperature and dissolved oxygen (DO) level were determined with a DO meter (HI 9147, Hanna Instruments, USA), pH with a LAB 850 set pH meter (Accumet AP115, USA), and water depth with a wooden ruler (2 m). Air temperatures at the sampling site were determined at the time of water sampling with a digital thermometer.

### Heavy metals analysis of plant tissue and soil

Plant and soil samples were dried at 70 °C for 3 days. Each sample was finely powdered to pass through a 250-μm mesh using an IKA mill. Rocks were dried at 110 °C for 24 h and then ground to a powder using an abrasion testing machine. The crushed rock material was sieved using a 75-μm mesh sieve. Plant material was placed in a vessel tube and digested with aqua regia (conc. 70% HNO_3_: 37% HCl = 1:3, v/v); the soil sample was digested with conc. 70% HNO_3_ and 30% hydrogen peroxide (H_2_O_2_), v/v; and fine crushed stones were digested with nitric acid (HNO_3_, Merck, Germany, trace metal grade) at different temperatures following the methods of APHA, AWWA, and WEF^56^. Digests were filtered through Whatman number 42 filter paper and brought to 25 mL with 1% HNO_3_ (trace metal grade). The water sample was filtered through a 0.45-μm membrane filter. Heavy metal (i.e., Cd, Cu, Ni, Cr, Zn, Pb, Fe, and Mn) concentrations were determined using FAAS. All standards were prepared with deionized (DI) water (resistivity 18.2 mΩ cm at 25 °C, Simplicity UV system, Millipore). Each sample was tested in triplicate and blank solutions were analyzed using identical methods in order to evaluate errors in analytical measurements. Standard test solutions were also analyzed after every 20 samples in order to obtain accurate, precise and reproducible results. NIST SRM 2710a Montana soil, JB-3 (basalt), and NIST SRM 1515 apple leaves were used as soil, rock and plant standard reference materials, respectively, for method validation. Percentage recoveries for the soil and plant materials were in the range of 98.2–108.3%, 94.3–100.7%, and 101.4–103.3% for different heavy metals, respectively. The relative standard deviation (RSD) ranged from 1.13–4.03%, 1.32–4.01% and 1.23–3.98% for soil, rock and plant materials, respectively.

### Diversity indexes and analyses

A 30-m transect along the stream of Huay Pha Lahd waterfall was used to establish 3 sampling plots (10 m × 10 m per plot). Each plot was divided into 100 sub-plots; the sub-plot was selected using random numbers (1 to 100); and four quadrate-frames (each 0.5 m × 0.5 m with 100 grid points) were laid on wet rocks (1-m^2^ sub-plot) to record percent cover of each taxon^57,58^.

The abundance of bryophytes in each sub-plot (Table S1) was estimated by using percent cover and 7 cover-class levels (0, 1, 2, …, 6 indicating 0, 1%, 5%, 25%, 75%, 95%, and 99%, respectively)^20,59^.

Species diversity was analyzed using relative cover (RC), relative frequency (RF) and importance value (IV)^20,60^. The indices of each taxon were calculated using the following equations:

$${\text{RC}} = {\text{C}}i/\Sigma {\text{C}} \times 100$$

where RC is the relative cover, C*i* is the percent cover of the *i*-th taxon, and ΣC is the total percent cover of all taxa.

$${\text{RF}} = {\text{F}}i/\Sigma {\text{F}} \times 100$$

where RF is the relative frequency, F*i* is the number of sub-plots that taxon *i*-th is present.

ΣF is the total occurrence of all taxa.

$${\text{IV}} = {\text{RF}} + {\text{RC}}$$

Species richness (s) was represented by the number of taxa in a sub-plot^20,61,62^.

Shannon–Wiener diversity index and Pielou’s evenness index were calculated using the following equations:

$${H}{^{\prime}}=-\sum_{i=1}^{s} {P}_{i} \ln {P}_{i}$$

where *H*′ is the Shannon–Wiener diversity index, *Pi* is the relative abundance of the *i*-th taxon, represented by relative cover.

$$J^{{\prime }} = H^{{\prime }} /\ln (s)$$

where *J*′ is Pielou’s evenness index, *H*′ is the Shannon–Wiener diversity index, *s* is the number of taxa.

Species area curve and Jackknife extrapolated numbers of taxa were performed using the PC-ORD software version 5 (MjM Software, Gleneden Beach, Oregon, USA), to represent percent sampling completeness from observed numbers of taxa^63–65^.

### Enrichment factors (EFs)

Enrichment factors were used to assess the contribution to metal content in bryophyte tissues from anthropogenic sources. Enrichment factors were calculated as follows, using the example of Fe:

$${\text{EFs}} = ({\text{C}}_{{\text{n}}} /{\text{C}}_{{{\text{Fe}}}} )_{{{\text{plant}}}} /({\text{C}}_{{\text{n}}} /{\text{C}}_{{{\text{Fe}}}} )_{{{\text{soil}}}}$$

where C_n_ is the concentration of the metal ‘n’ in bryophyte or soil samples, and C_Fe_ is the concentration of Fe determined before exposure. As proposed by Macedo-Miranda et al.^66^, *EF* was classified into four categories, *EF* ≤ 1, no contamination; 3 < *EF* < 5, slight contamination; 6 < *EF* ≤ 9, moderate contamination; and *EF* ≥ 10, highly contamination.

On a Windows-based PC, statistical analysis was performed using SPSS (SPSS, Chicago, IL). To identify significant differences in mean values, a one-way ANOVA and least significant difference (LSD) post hoc comparison were employed.

## Supplementary Information

Supplementary Information.

## Data Availability

The data that support the findings during the current study available from the corresponding author, WM on reasonable request.
