# Supplementary material for: The bryophyte community as bioindicator of heavy metals in a waterfall outflow
Source: Sci Rep. 2022 Apr 28;12:6942. doi: 10.1038/s41598-022-10980-9 (PMC9050711; doi:10.1038/s41598-022-10980-9)
Supplement: Supplementary file 1 — Supplementary Information. [file 41598_2022_10980_MOESM1_ESM.docx]

**Table S1.** Dataset of bryophyte percent cover and its cover class (in brackets) and codes of species name

| Subplot | 1_BRY | 2_CLA | 3_ECT | 4_FIS | 5_HYO | 6_MAR | 7_POR | 8_SCO |
| --- | --- | --- | --- | --- | --- | --- | --- | --- |
| P1_S48 | 2 (1) | 23 (2.5) | 26 (3) | 35 (3) | 3 (1.5) | 6 (2) | 0 (0) | 0 (0) |
| P1_S19 | 0 (0) | 0 (0) | 13 (2) | 24 (2.5) | 17 (2.5) | 0 (0) | 0 (0) | 0 (0) |
| P1_S33 | 0 (0) | 0 (0) | 0 (0) | 0 (0) | 0 (0) | 0 (0) | 0 (0) | 0 (0) |
| P1_S28 | 0 (0) | 0 (0) | 0 (0) | 18 (2.5) | 0 (0) | 0 (0) | 0 (0) | 0 (0) |
| P1_S46 | 0 (0) | 35 (3) | 13 (2) | 0 (0) | 0 (0) | 0 (0) | 0 (0) | 0 (0) |
| P1_S43 | 0 (0) | 0 (0) | 0 (0) | 0 (0) | 0 (0) | 0 (0) | 0 (0) | 0 (0) |
| P1_S84 | 0 (0) | 0 (0) | 0 (0) | 0 (0) | 19 (2.5) | 0 (0) | 14 (2) | 0 (0) |
| P1_S53 | 0 (0) | 0 (0) | 18 (2.5) | 0 (0) | 0 (0) | 0 (0) | 0 (0) | 5 (2) |
| P1_S74 | 7 (2) | 18 (2.5) | 23 (2.5) | 35 (3) | 5 (2) | 10 (2) | 0 (0) | 0 (0) |
| P1_S98 | 0 (0) | 0 (0) | 0 (0) | 24 (2.5) | 0 (0) | 0 (0) | 15 (2.5) | 0 (0) |
| P1_S24 | 0 (0) | 0 (0) | 0 (0) | 0 (0) | 0 (0) | 0 (0) | 0 (0) | 0 (0) |
| P1_S68 | 0 (0) | 0 (0) | 0 (0) | 0 (0) | 0 (0) | 0 (0) | 0 (0) | 0 (0) |
| P1_S14 | 4 (1.5) | 26 (3) | 15 (2.5) | 30 (3) | 0 (0) | 0 (0) | 0 (0) | 0 (0) |
| P1_S71 | 0 (0) | 0 (0) | 0 (0) | 0 (0) | 0 (0) | 0 (0) | 0 (0) | 0 (0) |
| P1_S38 | 0 (0) | 0 (0) | 0 (0) | 0 (0) | 0 (0) | 0 (0) | 0 (0) | 0 (0) |
| P2_S97 | 0 (0) | 0 (0) | 16 (2.5) | 19 (2.5) | 7 (2) | 0 (0) | 0 (0) | 0 (0) |
| P2_S69 | 0 (0) | 0 (0) | 0 (0) | 0 (0) | 0 (0) | 0 (0) | 0 (0) | 0 (0) |
| P2_S87 | 0 (0) | 0 (0) | 0 (0) | 41 (3) | 8 (2) | 0 (0) | 0 (0) | 0 (0) |
| P2_S57 | 0 (0) | 0 (0) | 21 (2.5) | 0 (0) | 10 (2) | 0 (0) | 0 (0) | 0 (0) |
| P2_S32 | 0 (0) | 0 (0) | 0 (0) | 0 (0) | 0 (0) | 0 (0) | 0 (0) | 0 (0) |
| P2_S10 | 0 (0) | 21 (2.5) | 0 (0) | 0 (0) | 16 (2.5) | 0 (0) | 17 (2.5) | 0 (0) |
| P2_S44 | 0 (0) | 0 (0) | 0 (0) | 0 (0) | 0 (0) | 0 (0) | 0 (0) | 0 (0) |
| P2_S04 | 0 (0) | 0 (0) | 22 (2.5) | 65 (3.5) | 0 (0) | 0 (0) | 0 (0) | 0 (0) |
| P2_S42 | 0 (0) | 0 (0) | 0 (0) | 0 (0) | 0 (0) | 0 (0) | 0 (0) | 0 (0) |
| P2_S73 | 0 (0) | 17 (2.5) | 0 (0) | 0 (0) | 0 (0) | 0 (0) | 0 (0) | 0 (0) |
| P2_S34 | 5 (2) | 0 (0) | 0 (0) | 0 (0) | 0 (0) | 0 (0) | 0 (0) | 0 (0) |
| P2_S25 | 0 (0) | 0 (0) | 35 (3) | 0 (0) | 0 (0) | 0 (0) | 0 (0) | 0 (0) |
| P2_S85 | 0 (0) | 0 (0) | 18 (2.5) | 23 (2.5) | 5 (2) | 0 (0) | 12 (2) | 0 (0) |
| P2_S79 | 0 (0) | 0 (0) | 0 (0) | 82 (4) | 13 (2) | 0 (0) | 0 (0) | 0 (0) |
| P2_S62 | 0 (0) | 32 (3) | 29 (3) | 0 (0) | 0 (0) | 0 (0) | 0 (0) | 0 (0) |
| P3_S90 | 0 (0) | 0 (0) | 0 (0) | 0 (0) | 0 (0) | 0 (0) | 0 (0) | 0 (0) |
| P3_S59 | 0 (0) | 0 (0) | 0 (0) | 0 (0) | 0 (0) | 0 (0) | 0 (0) | 0 (0) |
| P3_S08 | 0 (0) | 0 (0) | 20 (2.5) | 0 (0) | 2 (1) | 6 (2) | 0 (0) | 0 (0) |
| P3_S11 | 0 (0) | 0 (0) | 0 (0) | 0 (0) | 0 (0) | 0 (0) | 0 (0) | 0 (0) |
| P3_S46 | 0 (0) | 0 (0) | 0 (0) | 57 (3.5) | 0 (0) | 0 (0) | 0 (0) | 0 (0) |
| P3_S20 | 0 (0) | 0 (0) | 0 (0) | 0 (0) | 0 (0) | 0 (0) | 0 (0) | 0 (0) |
| P3_S48 | 0 (0) | 0 (0) | 56 (3.5) | 0 (0) | 0 (0) | 0 (0) | 0 (0) | 0 (0) |
| P3_S76 | 0 (0) | 25 (3) | 0 (0) | 33 (3) | 0 (0) | 0 (0) | 0 (0) | 0 (0) |
| P3_S39 | 0 (0) | 0 (0) | 0 (0) | 0 (0) | 0 (0) | 0 (0) | 0 (0) | 0 (0) |
| P3_S58 | 0 (0) | 0 (0) | 0 (0) | 0 (0) | 0 (0) | 0 (0) | 0 (0) | 0 (0) |
| P3_S72 | 0 (0) | 31 (3) | 42 (3) | 0 (0) | 0 (0) | 8 (2) | 0 (0) | 0 (0) |
| P3_S69 | 0 (0) | 0 (0) | 0 (0) | 0 (0) | 0 (0) | 0 (0) | 0 (0) | 0 (0) |
| P3_S01 | 0 (0) | 0 (0) | 0 (0) | 35 (3) | 6 (2) | 0 (0) | 0 (0) | 0 (0) |
| P3_S33 | 0 (0) | 0 (0) | 0 (0) | 0 (0) | 0 (0) | 0 (0) | 0 (0) | 0 (0) |
| P3_S82 | 2 (1) | 14 (2) | 35 (3) | 0 (0) | 3 (1.5) | 2 (1) | 0 (0) | 0 (0) |

Abbreviations: 1_BRY = *Bryum* sp.; 2_CLA = *Claopodium prionophyllum*; 3_ECT = *Ectropothecium zollingeri*; 4_FIS = *Fissidens crispulus* var. *crispulus*; 5_HYO = *Hyophila involuta*; 6_MAR = *Marchantia emarginata* var. *emarginata*; 7_POR = *Porella acutifolia* var. *birmanica*; 8_SCO = *Scopelophila cataractae*.
